# Total Recall: Intestinal T_RM_ Cells in Health and Disease

**DOI:** 10.3389/fimmu.2020.623072

**Published:** 2021-01-19

**Authors:** Eva-Maria Paap, Tanja M. Müller, Katrin Sommer, Markus F. Neurath, Sebastian Zundler

**Affiliations:** Department of Medicine 1 and Deutsches Zentrum Immuntherapie, University Hospital Erlangen, Friedrich-Alexander-Universität Erlangen-Nürnberg, Erlangen, Germany

**Keywords:** tissue-resident memory T cells, intestine, inflammatory bowel diseases, infection control, therapeutic targets

## Abstract

Tissue-resident memory T cells (T_RM_ cells) have crucial functions in host defense in mucosal tissues. They provide local adaptive immune surveillance and allow the fast initiation of targeted adaptive immune responses in case of antigen re-exposure. Recently, an aberrant activation in the case of immunologically mediated diseases has been increasingly acknowledged. As the organ with the largest interface to the environment, the gastrointestinal tract faces billions of antigens every day. Tightly balanced processes are necessary to ensure tolerance towards non-hazardous antigens, but to set up a powerful immune response against potentially dangerous ones. In this complex nexus of immune cells and their mediators, T_RM_ cells play a central role and have been shown to promote both physiological and pathological events. In this review, we will summarize the current knowledge on the homeostatic functions of T_RM_ cells and delineate their implication in infection control in the gut. Moreover, we will outline their commitment in immune dysregulation in gastrointestinal chronic inflammatory conditions and shed light on T_RM_ cells as current and potential future therapeutic targets.

## Introduction

Coordinated processes of the immune system require a tightly regulated interplay of various immune cell types and mediators. A particular feature of the adaptive immune system is the generation of immunological memory following antigen exposure leading to preparedness for the initiation of targeted immune responses in case of re-exposure. To this end, memory T cells are generated during a primary confrontation with an antigen. After its clearing, they survive as long-lived patrolling guards in particular compartments of the body.

Memory T cells are grouped into three main populations: central memory T cells (T_CM_), effector memory T cells (T_EM_), and tissue-resident memory T cells (T_RM_) (1–4). T_RM_ cells persist at epithelial surfaces including the gastrointestinal tract (GIT), skin, and lung as well as in non-barrier tissues such as the brain and the joints (3, 5–9). They are transcriptionally, phenotypically, and functionally distinct from recirculating central and effector memory T cells (10). Due to their localization at the interface between the host and the environment, they provide local adaptive immune surveillance for intruding cognate antigens, positioning them in the driver’s seat for the re-initiation of immune responses to known antigens in mucosal tissues (11). The GIT disposes over the largest surface of the body exposed to the external environment. This environment has a challenging composition including commensal, pathobiontic and sometimes pathogenic bacteria, viruses and, parasites as well as nutritional and potentially toxic antigens. Therefore, a closely regulated local immune system balancing tolerance and protection is essential and, as the first line of adaptive defence, T_RM_ cells play a key role in this context. This said, it is obvious that in addition to crucial functions in infection control, dysregulation of T_RM_ networks may also contribute to the development of diseases such as chronic inflammatory bowel diseases (IBD).

However, the role of T_RM_ cells in the intestine is not completely understood. In the following paragraphs, we will review the current knowledge on their implication in intestinal immune processes and also outline the putative contribution to pathological conditions as well as translational approaches to target T_RM_ cells.

## Phenotype of Intestinal T_RM_ Cells

T_RM_ cells have first been described in 2009 (4) and, early on, a specific profile of molecules associated with a T_RM_ phenotype was evident. More recently, Kumar and colleagues described a transcriptional and phenotypic signature that defines both CD8^+^ and CD4^+^ T_RM_ cells in humans and that is conserved across individuals and in mucosal and lymphoid tissues (12).

In general, the membrane protein CD69 is used to define both CD8^+^ and CD4^+^ T_RM_ cells. CD69 is a type II C-lectin receptor, which regulates, on the one hand, the differentiation of regulatory T cells and the secretion of cytokines like IL-17, IL-22, and interferon-γ (IFN-γ) and suppresses, on the other hand, the sphingosine-1-phosphate receptor 1 (S1PR1) [(13, 14), reviewed in (15)]. Mechanistically, CD69 interferes with the cell surface expression and function of S1PR1, which is essential for T and B cell egress from peripheral tissues, secondary lymphoid organs and thymus *via* chemotaxis towards S1P, which is present in high concentrations in the bloodstream (13, 16, 17). Moreover, a decreased expression of the transcription factor KLF2 in T_RM_ cells leads to the downregulation of S1PR1 (18). Together, the upregulation of CD69 and the downregulation of KLF2 and S1PR1 promote tissue retention of T_RM_ cells.

However, there is also evidence that CD69 is not expressed on all T_RM_ cells and—depending on the tissue—is not necessary for their generation. According to these studies, CD69 plays no discernible role for T_RM_ cell formation in the small intestine, while it is essential for T_RM_ cell development in the kidney in mice (19, 20).

Another important marker of T_RM_ cells is CD103, also called αE integrin. CD103 pairs with the β7 integrin chain and the heterodimer binds to E-cadherin, which is expressed on epithelial cells (21). Thus, this interaction constitutes an independent mechanism promoting mucosal retention. It was already shown in humans and in mice that the expression of CD103 is more predominant in CD8^+^ T_RM_ cells than in CD4^+^ T_RM_ cells (22–24). Moreover, in the human intestine, CD103 is not necessary for the persistence of CD4^+^ and CD8^+^ T_RM_ cells (6, 7, 22). Bergsbaken and colleagues even identified a preferential development of CD103^-^ T_RM_ cells in inflammatory microenvironments within the mouse *lamina propria* upon infection with *Yersinia pseudotuberculosis* (Yptb) (22).

Further core phenotypic markers for human CD8^+^ T_RM_ cells in multiple mucosal and lymphoid tissues include CD49a, CD101, and PD-1 (12), whereas CD161, a C-type lectin-like receptor seems to be specific for CD8^+^ T_RM_ cells in the human gut (25, 26). Furthermore, the T_RM_-specific gene signature includes the downregulation of lymph node homing molecules such as CD62L and CCR7, the upregulation of specific adhesion molecules like CRTAM, as well as the modulation of specific chemokine receptors including an increased CXCR6 and decreased CX3CR1 expression (12).

Several transcription factors have been implicated in the transcriptional control of T_RM_ cells leading to the expression of the above-mentioned molecules. In particular, Hobit together with Blimp-1 (PRDM1), Runx3, and Notch regulate the differentiation and maintenance of T_RM_ cells. Importantly, Hobit and Blimp-1 are known to synergistically control the expression of T_RM_ cell-regulated genes like CD69, KLF2, and S1PR1 (27–29). In this context, it is important to mention that Hobit expression is restricted to tissue-resident T cells [including T_RM_ cells, NKT cells, and some MAIT cells] in mice (27, 30), but not in humans. There, Hobit expression is also found in other T cell subsets with cytotoxic phenotype (31, 32).

Importantly, several cytokines like IL-15, IL-33, transforming growth factor-β (TGF-β), and tumor necrosis factor-α (TNF-α) were identified to play a role in the maintenance of T_RM_ cells (18, 33).

## T_RM_ Cells in Intestinal Infection Control

Especially in the GIT, T_RM_ cells are important in mediating fast and effective immune responses, when necessary. Thus, they crucially contribute to the maintenance of the local tissue homeostasis.

**Figure 1:**
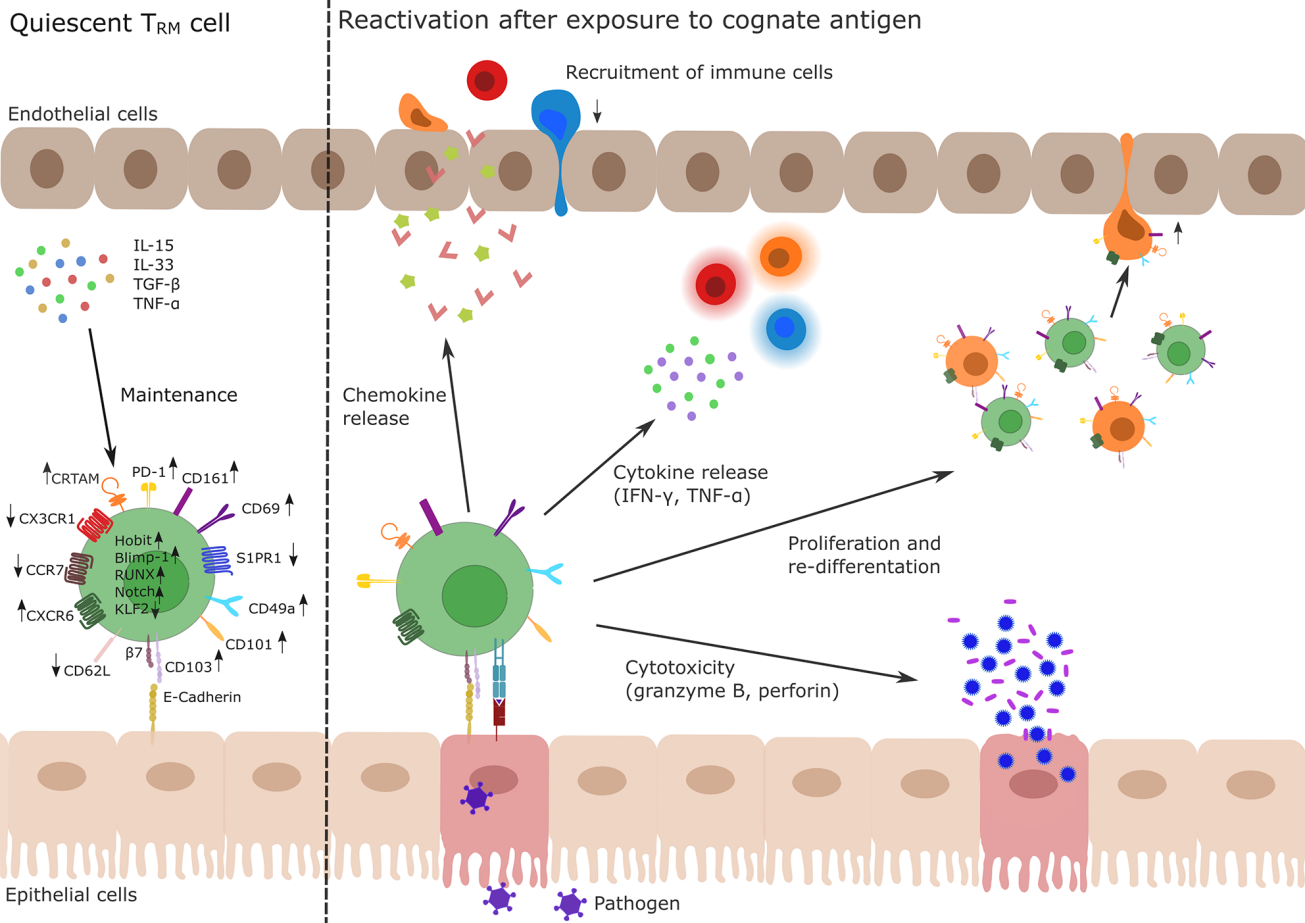
Profile and function of T_RM_ cells. Left side: T_RM_ cells develop during primary infection. The differentiation and maintenance of T_RM_ cells is controlled by tissue-derived signals, e.g., TNF-α, TGF-β or IL-15 and IL-33 resulting in the up- and down-regulation of different genes *via* activity of the transcription factors Hobit, Blimp-1, Runx3, and Notch and the silencing of Klf2. In particular, upregulation of CD69 and CD103 and simultaneous downregulation of S1PR1 are key drivers of T_RM_ cell tissue retention. Other membrane molecules highly expressed in T_RM_ cells are CD49a, CD101, PD-1, CRTAM, and CXCR6 while CD62L, CCR7, and CX3CR1 show a decreased expression pattern in T_RM_ cells. Right side: After re-exposure to a cognate antigen (e.g., from a pathogen, shown in purple), T_RM_ cells are able to initiate a fast immune response. This includes chemokine release to recruit lymphocytes (indicated as red, orange, and blue immune cells) to the site of infection, release of pro-inflammatory cytokines (IFN-γ, TNF-α) to activate other cells as well as the production of the cytotoxic effectors perforin or granzyme B. There is also evidence for the ability of T_RM_ cells to proliferate or to re-differentiate (indicated as green and orange cells) and to leave the tissue (orange ex-T_RM_ cells; for details cf. main text). T_RM_, tissue-resident memory T cell; TNF, tumor necrosis factor; TGF, transforming growth factor; IL, Interleukin; KLF, Krüppel-like factor; CD, cluster of differentiation; S1PR1, sphingosine-1-phosphate receptor 1; PD-1, programmed cell death protein 1; CRTAM, cytotoxic and regulatory T-cell molecule; CXCR, CXC-motif chemokine receptor; CCR, Chemokine receptor.

During primary infection, whether viral, bacterial or parasitic, some memory T cells acquire a T_RM_ phenotype including differential protein expression as described above and are retained in the tissue, where they are able to survive long-term (4, 34, 35). There seems to be considerable heterogeneity in intestinal T_RM_ populations as recently suggested by two studies building on single-cell transcriptomics in mice (36, 37). After re-infection with a previously encountered pathogen, the presence of T_RM_ cells provides a short-cut with regard to the time-consuming processes involved in *de-novo* adaptive immune responses, i.e. antigen processing by antigen-presenting cells (APCs), APC migration to secondary lymphoid tissues, T cell recognition, co-stimulation with subsequent activation, and proliferation as well as recirculation and migration of effector T cells to the infected tissue [reviewed in (38–41)]. Instead, upon antigen binding, T_RM_ cells are directly able to proliferate, to secrete pro-inflammatory cytokines such as IFN-γ or TNF-α and chemokines and to mediate cytotoxicity by secreting granzyme B and perforin to directly eliminate infected cells (**Figure 1**) [(5–7, 42), reviewed in (43)].

Interestingly, T_RM_ cells are not only generated at the site of primary infection but also seed distant locations. However, as shown by Sheridan and colleagues in mice, intestinal CD8^+^ T_RM_ cells developing upon oral infection with *Listeria monocytogenes* are more robust and have another phenotype than intestinal T_RM_ cells developing upon intranasal or intravenous infection (44).

Due to the increased abundance of CD8^+^ T_RM_ cells compared with CD4^+^ T_RM_ cells, the former have been examined in much more detail in the context of intestinal infections. Yet, CD4^+^ and CD8^+^ T_RM_ cells share several similarities and CD4^+^ T_RM_ cells crucially contribute to recall immunity by chemokine secretion and immune cell activation (45).

In summary, these observations suggest that T_RM_ cells might be important effectors of vaccination strategies in the gut. Consistently, a recent study showed that an oral typhoid vaccine was able to induce antigen-specific CD4^+^ T_RM_ cells in the human small intestine (46). Additionally, transient microbiota depletion-boosted immunization in mice has been proposed as a strategy to optimize T_RM_ cell generation upon exposure with vaccine antigens (47).

Studies by Bartolomé-Casado et al. revealed that both CD4^+^ and CD8^+^ T_RM_ cells persist for years in the human small intestine. Both undergo tissue-specific changes, which make them polyfunctional T_H_1 and T_C_1 cells (6, 7). How this longevity of T_RM_ cells is ensured is not completely elucidated so far and the question arises whether the size of the T_RM_ population in a homeostatic state is regulated by a continuous supply of recirculating memory T cells or whether a well-balanced T_RM_ cell proliferation is sufficient for the maintenance of the T_RM_ cell population [reviewed in (43)]. However, low-level homeostatic cell proliferation has been described for T_RM_ cells, e.g. in the skin and female reproductive tract, but not for the GIT so far (5, 48).

In contrast to the view that T_RM_ cells are confined within “their” tissue, Fonseca and colleagues showed that there is also evidence for fully differentiated T_RM_ cells in mice, which re-differentiate and recirculate into lymphoid tissues (49). Moreover, it was shown that CD4^+^ T_RM_ cells in the skin may have the ability to downregulate CD69 and subsequently exit the tissue (50). Very recently, this has been demonstrated for intestinal CD8^+^ T_RM_ cells following oral *Listeria monocytogenes* re-infection. Using a Hobit reporter mouse strain, Behr and co-workers could elegantly show that ex-T_RM_ cells appeared in the circulation and were able to mount systemic and local immune responses (51).

Taken together, these data show that T_RM_ cells represent an important switch point in recall immunity. However, the presence of this cell type, which is able to mediate powerful immune responses also entails the risk that dysregulation and imbalance can lead to immune dysfunctions like allergic disorders or chronic inflammation.

## T_RM_ Cells in Inflammatory Bowel Diseases

In recent years, the implication of T_RM_ cells in pathological conditions has been increasingly acknowledged. In particular, they seem to play an important role in various cancer entities and several immune-mediated inflammatory disorders like psoriasis, vitiligo, psoriatic arthritis, and IBD (52–58). Whereas T_RM_ cells as tumor-infiltrating lymphocytes (TIL) are associated with a better prognosis in most cancer types (e.g. ovarian cancer, breast cancer, and gastric adenocarcinoma), CD103^+^ TIL in colorectal cancer are associated with poor prognosis (56–59), suggesting that their impact is tissue-specific.

In the context of IBDs, an important role of T_RM_ cells has only recently emerged. Several studies indicate that the presence and generation of T_RM_ cells are involved in the pathogenesis of IBDs (**Table 1**). We were able to show that CD69^+^CD103^+^ cells with a T_RM_ phenotype are increased in the lamina propria of patients with ulcerative colitis (UC) and Crohn’s disease (CD) and that high levels of CD4^+^ T_RM_ cells in IBD patients are associated with early relapse. In mice, we observed that the key T_RM_ transcription factors Hobit and Blimp-1 are essential for experimental colitis since their absence protected from T cell transfer colitis, dextran sodium sulphate-induced colitis and trinitrobenzene sulfonic acid-induced colitis. Mechanistically, we could attribute this to an adaptive-innate crosstalk mechanism including chemokine release by T_RM_ cells and subsequent recruitment and differentiation of pro-inflammatory immune cells (55). Consistent with these results Bishu and colleagues reported, that CD4^+^ T_RM_ cells are increased in CD compared with control patients and identified these CD4^+^ T_RM_ cells as the major T cell source of TNF-α in the mucosa of CD patients. Furthermore, these cells produced more IL-17A and TNF-α in inflamed compared to healthy tissue (60). Bottois and colleagues profiled two distinct CD8^+^ T_RM_ cell subsets in CD, defined by KLRG1 and CD103, which are both receptors of E-Cadherin. CD103^+^CD8^+^ T_RM_ cells in CD patients expressed T_H_17-related genes such as CCL20, IL-22 and, IL-26 suggesting that they may trigger innate immune responses as well as the recruitment of effector cells. KLRG1^+^CD8^+^ T_RM_ cells were specifically elevated under inflammatory conditions and showed increased proliferative and cytotoxic potential (61). Furthermore, a recent study employing single-cell RNA-sequencing identified changes in the transcriptional profile of CD8^+^ T_RM_ cell subsets in UC including a pro-inflammatory phenotype and increased expression of Eomesodermin (62). Similarly, Corridoni and colleagues reported that CD8^+^ T_RM_ cells in UC express more GZMK and IL26, suggesting that altered CD8^+^ T_RM_ cells are implicated in UC pathogenesis (63).

**Table 1:** Overview of studies on the role of T_RM_ cells in IBD.

Organsim	Key conclusions on T_RM_ cells	Ref.
**Human and Mouse**	Human: → CD69^+^CD103^+^ cells with a T_RM_ phenotype are increased in the lamina propria of patients with ulcerative colitis (UC) and Crohn’s disease (CD) → High levels of CD4^+^ T_RM_ cells in IBD patients are associated with early relapse. Mouse: → T_RM_ cells expressing Hobit and Blimp-1 are key drivers of experimental colitis due to an adaptive-innate crosstalk mechanism	(55)
**Human**	→ Increased CD4^+^ T_RM_ cell population in CD compared with control patients → Increased production of IL-17A and TNF-α by T_RM_ cells in inflamed compared to healthy tissue → Major T cell source of TNF-α in the mucosa of CD patients.	(60)
**Human**	→ Two distinct CD8^+^ T_RM_ cell subsets in CD, defined by KLRG1 and CD103 → CD103^+^CD8^+^ T_RM_ cells: express T_H_17-related genes such as CCL20, IL-22, and IL-26 → KLRG1^+^CD8^+^ T_RM_ cells: specifically elevated under inflammatory conditions, show increased proliferative and cytotoxic potential	(61)
**Human**	→ Changes in the transcriptional profile of CD8^+^ T_RM_ cell subsets in UC: pro-inflammatory phenotype and increased expression of Eomesodermin	(62)
**Human**	→ CD8^+^ T_RM_ cells in UC express more GZMK and IL26 → Altered CD8^+^ T_RM_ cells may be implicated in UC pathogenesis	(63)
**Human**	→ Reduced numbers of CD103^+^Runx3^+^ T_RM_ cells with a probably regulatory phenotype in CD and UC: expression of CD39 and CD73, release of IL-10	(64)
**Human**	→ Decreased numbers of CD103^+^CD4^+^ and CD103^+^CD8^+^ T cells in active IBD → Rise of the numbers of these cells in the remission phase up to levels comparable with healthy controls.	(65)

Yet, observations made by other groups support the notion that the picture is more complex. E.g., Noble et al. described reduced numbers of CD103^+^Runx3^+^ T_RM_ cells in CD and UC. They observed the expression of CD39 and CD73 on these cells as well as the release of IL-10 suggesting that these cells have a regulatory phenotype. They hypothesized that T_RM_ cells probably serve as gatekeepers by controlling the access of mucosal antigens to germinal centers in lymphoid tissue (64). Roosenboom and colleagues reported decreased numbers of CD103^+^CD4^+^ and CD103^+^CD8^+^ T cells in active IBD and found a rise of these numbers in the remission phase up to levels comparable with healthy controls. In addition, they observed a lower number of CD103^-^ T cells in healthy controls and IBD patients in remission in comparison with active CD and UC patients (65). Importantly, this study was not specifically designed to assess T_RM_ cells. Thus, it seems possible that these data are actually indicative of a change in T_RM_ cell phenotype similar to some of the studies mentioned above.

Taken together, T_RM_ cells are undoubtedly involved in the pathogenesis of IBDs. However, different observations have been made with regard to their function and mechanisms. While these seem to be conflicting on first view, it is likely that they rather derive from different approaches to a complex issue. For example, considering that T_RM_ cell generation may occur following any recognition of a cognate antigen by a naïve T cell, it is also clear that—depending on co-stimulatory signals and the nature of the surrounding environment—different forms of T cell memory may be imprinted. Thus, it is not surprising that regulatory as well as pro-inflammatory T_RM_ phenotypes have been described depending on the markers chosen to identify the cells. In consequence, the reduction of regulatory-type T_RM_ cells is actually not at all contradicting other observations, such as perturbed T_RM_ cell phenotypes in IBD or increased pro-inflammatory T_RM_ cell populations. Yet, further investigations are necessary to answer the remaining open questions.

## T_RM_ Cells as Potential Therapeutic Targets in Inflammatory Bowel Diseases

Based on the above-mentioned reports T_RM_ cells seem to be a promising therapeutic target to treat UC and CD.

Specific approaches in that regard are still lacking and would require the identification of unique targets on or in T_RM_ cells as well as the selection of appropriate targeting strategies. However, the mechanism of the monoclonal anti-β7 integrin antibody etrolizumab, which blocks the αEβ7 and α4β7 integrin heterodimers might in part be explained by effects on T_RM_ cells. For example, this antibody has been shown to block the retention of CD8^+^ T cells from patients with UC in a humanized *in vivo* cell trafficking model suggesting that it might also reduce the retention of T_RM_ cells in the gut (66). Moreover, *post-hoc* analyses of the successful phase II trial in UC showed that patients with high expression of CD103 were more likely to respond to etrolizumab therapy (67, 68). Etrolizumab recently completed an ambitious phase III trial program in UC, in which only two out of three induction trials and no maintenance trial reached the primary endpoint. However, the drug was efficient in several important secondary endpoints and was similarly effective as infliximab and adalimumab, underscoring its biological activity and warranting further research (69–72). Phase III trials in CD are still ongoing with promising results in an exploratory cohort (73, 74).

As mentioned above, the downregulation of S1PR1 is a hallmark of T_RM_ cells. In this context, it is tempting to speculate, which effect the class of S1PR modulators including ozanimod, etrasimod, and amiselimod, which are currently also investigated for application in IBDs might have on intestinal T cells (75, 76). While it is evident that they lead to sequestration of naïve T cells and T_CM_ cells in secondary lymphoid organs (77), one could also assume that they reduce recirculation of T cells from the tissue driving the retention of local non-T_RM_ T cells.

Some of the drugs already in use in IBD might also partly affect T_RM_ cells in the gut. For instance, the anti-α4β7 integrin antibody vedolizumab that blocks T cell homing to the gut *via* MAdCAM-1 might reduce the recruitment of pre-T_RM_ cells and, thus, prevent the seeding of new T_RM_ cells [reviewed in (78)]. The anti-IL-12/23 antibody ustekinumab is thought to block the generation and differentiation of T_H_1 and T_H_17 cells [reviewed in (79)]. This will certainly also affect T_RM_ cells with a T_H_1 or T_H_17 phenotype, e.g. the *de-novo* generation of such cells might be reduced or established T_RM_ cells might be subjected to plasticity due to an altered cytokine balance (80, 81). Another drug routinely used in UC is tofacitinib, which inhibits the Janus kinase (JAK) pathway (mainly JAK1 and JAK3) and, thus, abrogates signaling of numerous cytokines (82, 83). This also affects IL-15, which is known to participate in the maintenance of T_RM_ cells (18, 33, 84). In the skin, it has already been shown that targeting CD122, a subunit of the IL-15 receptor, is a potential treatment strategy for tissue-specific autoimmune diseases involving T_RM_ cell such as vitiligo (85).

Collectively, research on T_RM_ cells as a therapeutic target is still in its infancy. However, several currently used and developed drugs, particularly etrolizumab and S1PR1 modulators, might interfere with T_RM_ cells and it is likely that the coming years will reveal further details on their suitability for treating IBD.

## Concluding Remarks

Over the last decade, T_RM_ cells have emerged as an important cell population in mucosal tissues controlling the initiation of secondary immune responses. Multiple efforts have led to a precise characterization of their phenotype and implication in infection control. Moreover, they have been increasingly associated with pathological conditions, in the case of the GIT, particularly with IBD. Although not all questions are already resolved, T_RM_ cells seem to control important steps in the pathogenesis of chronic intestinal inflammation and, thus, represent a potential target for future IBD therapy. Further research is necessary to better define their pathogenetic contributions and to develop targeted therapeutic approaches.

## Author Contributions

E-MP, TM, KS, MN, and SZ wrote the manuscript. All authors contributed to the article and approved the submitted version.

## Funding

German Research Foundation (DFG, ZU 377/4-1), Interdisciplinary Center for Clinical Research, University Hospital Erlangen (A84). We acknowledge support by Deutsche Forschungsgemeinschaft and Friedrich-Alexander-Universität Erlangen-Nürnberg (FAU) within the funding program Open Access Publishing.

## Conflict of Interest

SZ and MN received research support from Takeda, Roche, and Shire. MN has served as an advisor for Pentax, Giuliani, MSD, Abbvie, Janssen, Takeda, and Boehringer.

The remaining authors declare that the research was conducted in the absence of any commercial or financial relationships that could be construed as a potential conflict of interest.
